# Skull and vertebral bone marrow in central nervous system inflammation

**DOI:** 10.1016/j.fmre.2023.01.012

**Published:** 2023-03-04

**Authors:** Honglei Ren, Qiang Liu

**Affiliations:** Department of Neurology, Tianjin Neurological Institute, Tianjin Institute of Immunology, State Key Laboratory of Experimental Hematology, International Joint Laboratory of Ocular Diseases, Ministry of Education, Haihe Laboratory of Cell Ecosystem, Tianjin Medical University General Hospital, Tianjin 300052, China

**Keywords:** Central nervous system, Bone marrow, Hematopoiesis, Immune cells, Neuroinflammation, Stroke, Multiple sclerosis

## Abstract

Emerging evidence has highlighted the capacity of hematogenous cells in skull and vertebral bone marrow to enter the meningeal borders via ossified vascular channels and maintain immune homeostasis in the central nervous system (CNS). CNS-adjacent skull and vertebral bone marrow comprises hematopoietic niches that can sense CNS injury and supply specialized immune cells to fine-tune inflammatory responses. Here, we review recent advances in our understanding of skull and vertebral bone marrow-derived immune cells in homeostasis and inflammatory CNS diseases. Further, we discuss the implications for future development of therapies to mitigate CNS inflammation and its detrimental sequelae in neurological disorders.

## 1 Introduction

The conventional view suggests that adult hematopoiesis in bone marrow is the source of immune cells that circulate in the blood and survey the peripheral organs. The hematopoietic system harbors cells capable of proliferating and differentiating to meet the demands of blood and immune cell production for maintaining homeostasis. All blood cells are derived from hematopoietic stem and progenitor cells (HSPCs), which reside predominantly in the bone marrow and are capable of self-renewal and differentiation into various immune cell types. These bone marrow-derived immune cells can swiftly respond to danger signals and mount an immune response against systemic stress, infections, and ischemia [1,2].

At unique loci skull and vertebral bone marrow surrounds the central nervous system (CNS) and houses blood cells that can sense and participate in CNS inflammation. Recent advances have revealed the presence of ossified vascular channels that connect the skull to the meninges, allowing immune cells derived from bone marrow niches to enter the meningeal borders [3], [4], [5]. Together with emerging evidence of the activation of bone marrow myeloid cells in neurological disorders such as stroke and multiple sclerosis (MS) [6], [7], [8], these studies suggest that the skull and vertebral bone marrow is an active player in the progression of CNS inflammation. Herein, we review growing evidence that skull and vertebral bone marrow-derived immune cells have key roles in neuroimmune interactions that govern the progression and resolution of CNS inflammation.

## 2 Anatomy of skull and vertebral bone marrow

**Fig. 1:**
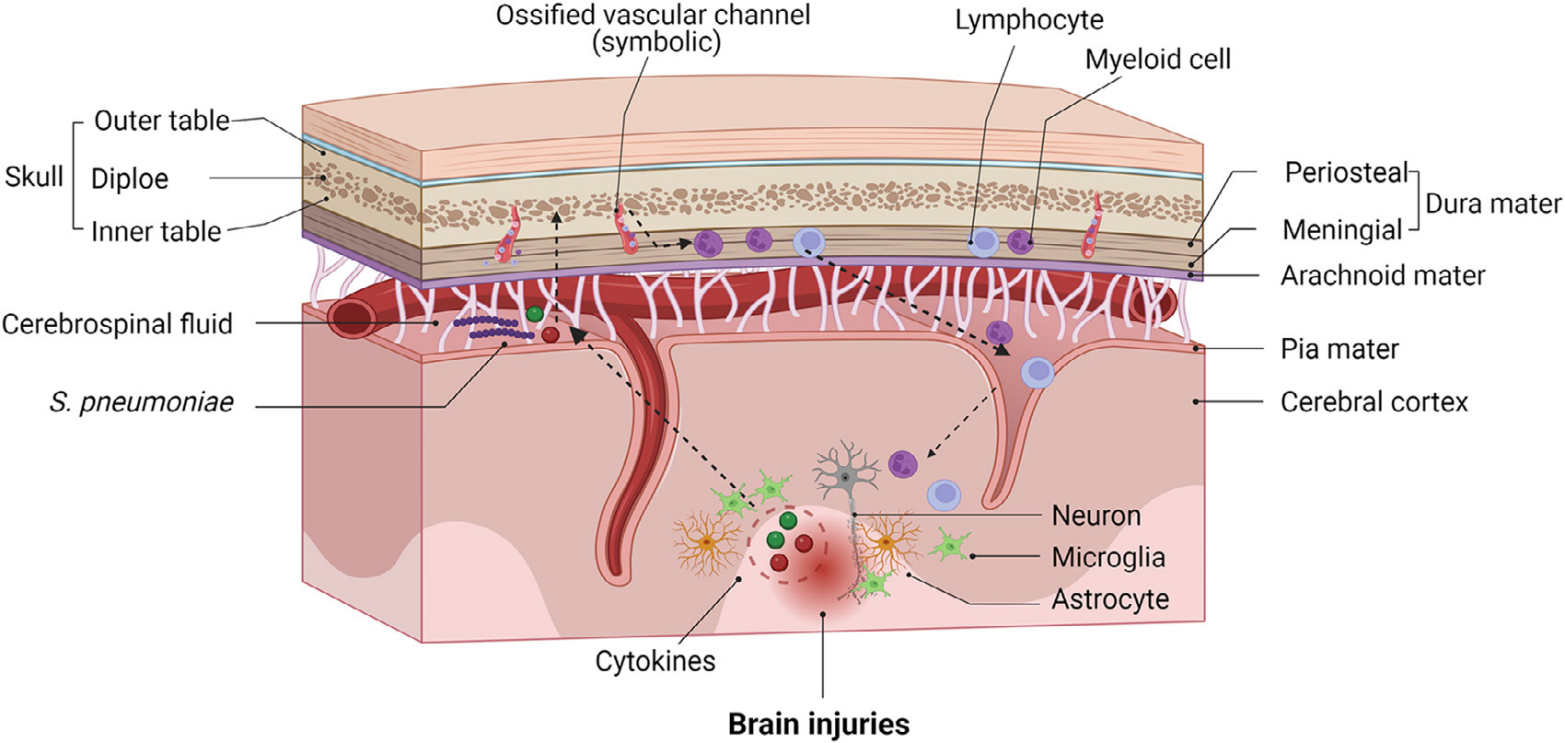
**Skull bone marrow and its connectivity to the meninges and parenchyma**. The skull surrounds the central nervous system and harbors hematopoietic stem and progenitor cells in the skull bone marrow. The bone marrow is connected to the dura mater by direct ossified vascular channels through which myeloid cells and lymphocytes enter the parenchyma, bypassing the blood-brain barrier. Cerebrospinal fluid can carry cues into the marrow via dural vascular channels to instruct the supply of skull bone marrow-derived immune cells.

The brain and spinal cord are surrounded by the skull and vertebral bones. According to shape classification, the skull is among the flat bones, whereas the vertebrae are irregular bones. The bones of the skull consist of the inner and outer cortical tables and the diploe or marrow space between them. This diploe houses the perivascular HSPC niche and receives stromal support from mesenchymal stem (MSCs) and endothelial cells [9]. The meninges are a three-layer membrane structure located underneath the skull bones [10]. The outermost layer, the dura mater, is a thick membrane composed of the periosteal layer, which is located in close proximity to the inner skull, and the meningeal layer, which is encased by flattened cells and located closer to the brain [11]. The middle meninx, the arachnoid mater, is a thin, transparent membrane that surrounds the brain and spinal cord like a loose-fitting sac. The arachnoid mater comes into direct contact with the dura mater but is separated from the pia mater, the innermost layer of the meninges, by the subarachnoid space. This space contains the cerebrospinal fluid (CSF) [12], which constitutes the major route for immune surveillance of the brain and is predominantly composed of T cells recruited through α4β1 integrin and C—X—C chemokine receptor 3 [13]. Ultimately, up to 15% of the CSF drains into the lymphatics within the perineural spaces of the cranial and spinal nerves. The cells of the skull and vertebral bone marrow are highly proliferative in adults. Recent studies in mice and humans have demonstrated direct short vascular channels connecting the cranial bone marrow to the meninges [3,14], potentially serving as a portal into the CNS for hematogenous cells derived from the surrounding bone marrow niches (Fig. 1). Therefore, the skull and vertebral bone marrow may provide a unique supply of hematogenous cells to the CNS in health and disease (Box 1). These findings highlight the apparent role of these cells as an intermediate between CNS resident and non-resident immune cells [15].

**Box 1. Major findings on the structure and function of skull and vertebral bone marrow.**

• Direct vascular channels exist between skull bone marrow and the brain surface (2018).
• Meningeal monocytes and neutrophils are derived from adjacent skull bone marrow (2021).
• Meningeal B cells migrate from skull bone marrow through specialized vascular connections (2021).
• Cerebrospinal fluid (CSF) accesses skull bone marrow via vascular channels and regulates skull bone marrow niches (2022).
• CSF exits into skull bone marrow and instructs hematopoiesis in bacterial meningitis (2022).

Alt-text: Unlabelled box

## 3 Skull and vertebral bone marrow as the neuroimmune interface

The CNS was initially defined as a site of immune privilege, since tissues that are rapidly rejected by the immune system when transplanted into the peripheral organs show prolonged survival when grafted into the CNS. Data accumulated over the past two decades has dramatically altered this viewpoint. The CNS not only undergoes constant immune surveillance in the meningeal compartment but also has functional lymphatic vessels lining the dural sinuses that carry CSF and accompanying immune cells to the deep cervical lymph nodes [16]. CNS-derived antigens in the CSF accumulate around the dural sinuses, are captured by local antigen-presenting cells, and are presented to patrolling T cells [17]. The CNS-associated bone marrow cavities and dura mater are linked by direct vascular channels through which marrow-derived myeloid cells can migrate to the adjacent brain tissue in the process of neuroinflammation [3,14]. Likewise, lymphocytes in the meninges and brain parenchyma, including B cells, are mainly derived from the surrounding bone marrow rather than the long bones or other immune organs in the periphery [4,5]. Hematopoiesis directly adjacent to the brain supplies myeloid cells and lymphocytes that bypass the blood-brain barrier. In addition, these early B cells may complete their maturation within the meningeal compartment, where dural fibroblast-derived factors such as C—X—C chemokine ligand 12 (CXCL12) can facilitate their development [4]. Substantiating these findings, one study showed that acute lymphoblastic leukemia cells could travel into the CNS along vessels that passed directly between the skull or vertebral bone marrow and subarachnoid space [18].

The ossified channels in the CNS-adjacent bones support two-way traffic: immune factors from the inflamed CNS may access the bone marrow to instruct HSPCs within the hematopoietic niche. Supporting this view, studies have reported direct CSF outflow to the skull bone marrow via these channels, carrying immune factors to the diverse hematopoietic and stromal cells within the niches [15,19] to fine-tune CNS immunity in MS or other neurological disorders [20] (Fig. 1). A recent study showed that arachnoid granulations are enriched with bone marrow–derived cells, implying their fundamental role as conduits of immune elements such as antigen-presenting cells that communicate with the bone marrow and dura-arachnoid stroma [21]. Finally, 48 h after intrathecal gadobutrol administration in patients with CNS disorders, tracer enrichment was observed within the diploe of the skull bone marrow near the parasagittal dura and remote from the dura extensions [22]. As an emerging interface of neuroimmune interactions, the skull and vertebral bone marrow deserves closer examination in future studies.

## 4 Neural regulation of the bone marrow microenvironment

MSCs are major stromal cells that orchestrate the homeostasis and activity of HSPCs, which develop in the bone marrow niche [23]. MSCs are primarily located in the perivascular space and are associated with arterioles or sinusoidal blood vessels; here, they produce niche factors such as CXCL12 and stem cell factor [9]. Single-cell RNA analysis of the bone marrow stroma has revealed multiple stromal cell subsets expressing distinct genes that regulate hematopoietic differentiation [24]. Cellular taxonomy of the bone marrow stroma highlights the role of these cells in the control of homeostasis and differentiation of HSPCs in health and disease [24,25]. One recent study characterized the transcriptional landscape of bone marrow vascular, perivascular, and osteoblast cell subsets and found that local signals, including vascular Notch delta-like ligand 4 regulation, are essential for myeloid differentiation [26]. Together with colony-stimulating factor 1, these hematopoietic factors are generated by niche MSCs to optimize systemic immunity by orchestrating leukocyte output [27], [28], [29], [30].

The differentiation balance of bone marrow HSPCs is tightly controlled by neurogenic innervation. Sympathetic innervation acts as a key switch in the HSPC niche by coordinating the release of adrenergic neurotransmitters into the microenvironment. Indeed, a decline in sympathetic innervation during aging can lead to an irregular output of myeloid cells from bone marrow HSPCs; moreover, the administration of a selective adrenergic receptor 3 agonist can act on MSCs to rejuvenate HSPCs and restore the balanced output of leukocytes [31]. Unlike sympathetic nerves that regulate HSPC indirectly via stromal cells, nociceptor neurons drive HSPC mobilization by producing calcitonin gene-related peptide, which acts directly on HSPCs [32]. Together, these findings highlight the pivotal role of neurogenic innervation in controlling HSPC mobilization.

## 5 Skull and vertebral bone marrow in CNS disorders

Stroke is a leading cause of death and disability worldwide. Approximately 87% of strokes are ischemic, while intracerebral hemorrhage (ICH) is less common but fatal. ICH causes immediate neural injury due to mass effect and perihematomal edema [33,34]. Acute brain injury activates the immune system and the transmigration of peripheral immune cells into the perivascular space and brain parenchyma, which amplifies neuroinflammation and neurovascular injury [35]. Among the earliest immune cells that infiltrate the brain, neutrophils contribute to neural injury by releasing proteases, including metalloprotease 9 and cathepsin G, reactive oxygen and nitrogen species, and inflammatory interleukin (IL)-1β [36]. Monocyte-derived macrophage inflow into the ischemic brain depends on C—C chemokine receptor 2 and peaks at day 3 after onset [37,38]. To exert immune regulatory effects, activated macrophages may express Arg1, YM1, and the C-type lectin mannose receptor. These cells also produce pro-inflammatory cytokines such as tumor necrosis factor alpha, IL-1β, and IL-6 [36,39]. Following myeloid cells, natural killer, T, and B cells arrive in the injured brain. Upon entry, these lymphocytes trigger a cascade of secondary events to catalyze brain injury by conditioning the focal inflammatory milieu and accelerating neurovascular dysfunction [37,38]. Bone marrow hematopoiesis replenishes peripheral immune cells and provides myeloid and lymphoid cells that control the progression of brain inflammation. In ischemic stroke, skull rather than long bone marrow supplies myeloid cells, such as neutrophils, to the damaged brain [3,40]. A significant decrease in stromal cell-derived factor 1 levels in skull bone marrow has been reported, which may, at least in part, contribute to the release of neutrophils and monocytes [3,41]. Following ICH, HSPCs in the skull bone marrow shift toward the myeloid cell lineage. A recent study revealed an increased output of bone marrow Ly6C^low^ monocytes infiltrating the ICH brain, where they generated alternatively-activated macrophages and suppressed inflammatory brain injury [7]. Notably, administration of the US Food and Drug Administration approved β3-adrenergic receptor agonist mirabegron stimulates the production of immunomodulatory monocytes in the bone marrow, reducing neuroinflammation and improving ICH outcomes. Furthermore, a recent report demonstrated that a heterogeneous population of myeloid progenitors in the leptomeninges appeared to be continuously recruited from elsewhere, supporting the theory that bone marrow-derived immune cell mobilization is involved in active CNS immune surveillance and neuroinflammation [42].

The roles of bone marrow and hematopoietic stem cells in MS have received increasing attention. For example, autologous hematopoietic stem cell transplantation has been used in drug-resistant progressive MS to effectively eliminate pathogenic autoreactive T and B cells and “reset” the immune system with “brand new” lymphoid and myeloid cells [43,44]. Because the skull and vertebral bone marrow supplies myeloid cells and lymphocytes to the meninges and CNS parenchyma [5], these niches and immune cells may be early responders that participate in CNS inflammation and autoimmunity. A recent study revealed that HSPCs within bone marrow, including that of the skull and vertebral bones, can sense immune activation through autoreactive T cells that can switch on myelopoiesis. Subsequently, neutrophils and Ly6C^high^ monocytes are generated via the C—C chemokine ligand 5/C—C chemokine receptor 5 axis, resulting in augmented T-cell autoimmunity and increased CNS demyelination [6]. These findings demonstrate that the bone marrow niche is a unique site for stimulating CNS inflammation and autoimmunity during MS progression [45]; however, the relative contributions of long versus CNS-associated bones to MS progression require further study.

Skull bone marrow supposedly supplies immune cells to the brain and regulates brain immunity in aging and age-related neurological disorders. Accordingly, a recent study showed the accumulation of age-associated B and plasma cells in the meninges during aging [4]. Meningeal mucosal-associated invariant T (MAIT) cells are innate-like T cells responsible for producing high levels of antioxidative molecules. MAIT-cell deficiency leads to the accumulation of reactive oxygen species in the meninges, resulting in the reduction of junctional proteins and meningeal barrier leakage, which contributes to cognitive impairment [46]. Although the cellular dynamics and the sources of these cells require further investigation, these findings emphasize CNS-associated bone marrow as a source of immune cells that may influence brain inflammation through the calvarial-meningeal path in neurodegenerative diseases.

## 6 Conclusion and future perspectives

**Fig. 2:**
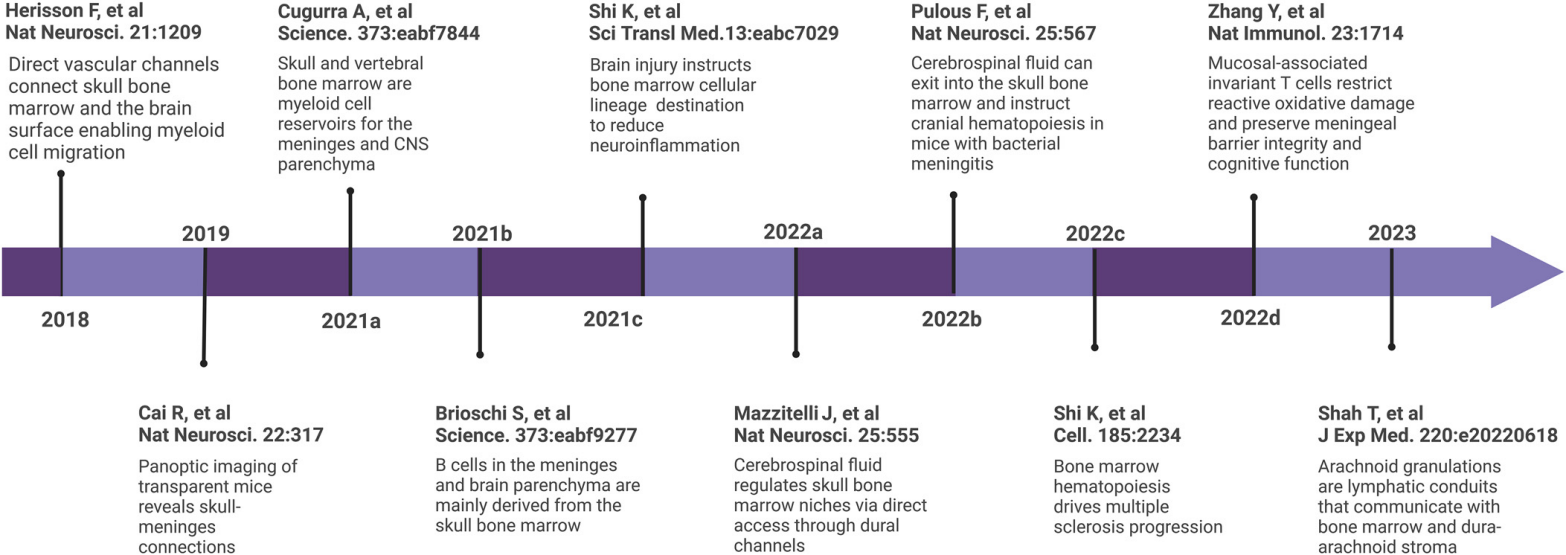
**Timeline of key skull and vertebral bone marrow and central nervous system discoveries**.

As the primary site of adult hematopoiesis, recent research has illustrated that the bone marrow rapidly senses immune activation in numerous neurological diseases and adapts through the production of new cells (Fig. 2). In this process, the skull and vertebral bone marrow and the dura mater connect via direct vascular channels through which newly produced hematogenous cells migrate to the meninges and parenchyma to orchestrate CNS inflammation.

The identification of aberrant myelopoiesis in the skull and vertebral bone marrow of patients with stroke and MS suggests that the bone marrow niche is a central hub for adaptation to immune activation and precise modulation of CNS inflammation. Although an increased output of monocytes and granulocytes occurs in stroke and MS, the consequences of myelopoiesis and its impact on CNS inflammation may vary. For example, bone marrow-derived Ly6C^low^ patrolling monocytes were reportedly preferentially increased in ICH, whereas inflammatory monocyte and neutrophil production was predominantly augmented in MS [6,7]. This discrepancy is likely caused by different signals received by the bone marrow niche. In stroke, bone marrow cells are activated due to increased adrenergic input via the β3 receptor; conversely, autoreactive T cells homing to the bone marrow niche trigger bone marrow cell activation in MS.

Although the ability of cells within the marrow of CNS-adjacent bones to sense and adapt to inflammatory stimuli makes them central in CNS inflammation, several major questions remain unanswered (Box 2). First, the dynamics of CNS-associated bone marrow cell activity over the course of neurological diseases awaits detailed characterization. Lineage tracing in combination with single-cell sequencing could provide valuable information regarding the cellular and molecular alterations of bone marrow during CNS injury and recovery. Second, the precise signals that dictate CNS bone marrow cell activity remain unclear. Multi-omics approaches using patient samples may provide new insight into the underlying mechanisms. Third, CNS injury and inflammatory signals may epigenetically imprint an “inflammatory memory” in bone marrow cells, thereby contributing to trained immunity. Investigating whether and how trained immunity in the bone marrow niche contributes to CNS inflammatory diseases would be intriguing. Finally, bone marrow hematopoiesis is governed by a specialized niche microenvironment comprising mesenchymal stromal cells, endothelial cells, and megakaryocytes. It remains unclear to what extent and by what mechanisms these components regulate bone marrow hematopoietic activity in the context of neurological disorders. These emerging questions must be addressed by interdisciplinary studies integrating expertise in immunology, hematology, proteomics/genomics and neurology. Such research could facilitate the design of new immunotherapies for inflammatory neurological disorders to reset aberrant bone marrow immunity and restore immune homeostasis.

**Box 2. Unanswered questions about skull and vertebral bone marrow in central nervous system inflammation.**

• What are the dynamics of cells in the central nervous system (CNS)-surrounding bone marrow over the course of various neurological diseases?
• What are the precise signals that instruct bone marrow cell activity within CNS-associated bones in neurological diseases?
• Do CNS insults imprint an “inflammatory memory” to influence disease outcomes?
• How do bone marrow stromal cells adapt to CNS insults and instruct hematopoietic activity?
• What are the common and distinct features of skull and vertebral bone marrow niches?

Alt-text: Unlabelled box

## Declaration of competing interest

The authors declare that they have no conflicts of interest in this work.
